# Mathematical Analysis of Glioma Growth in a Murine Model

**DOI:** 10.1038/s41598-017-02462-0

**Published:** 2017-05-31

**Authors:** Erica M. Rutter, Tracy L. Stepien, Barrett J. Anderies, Jonathan D. Plasencia, Eric C. Woolf, Adrienne C. Scheck, Gregory H. Turner, Qingwei Liu, David Frakes, Vikram Kodibagkar, Yang Kuang, Mark C. Preul, Eric J. Kostelich

**Affiliations:** 1 School of Mathematical and Statistical Sciences, Arizona State University, Tempe, AZ 85287 USA; 2 Center for Research in Scientific Computation, North Carolina State University, Raleigh, NC 27695 USA; 3 Department of Mathematics, Univeristy of Arizona, Tucson, AZ 85721 USA; 4 School of Biological and Health Systems Engineering, Arizona State University, Tempe, AZ 85287 USA; 5 School of Life Sciences, Arizona State University, Tempe, AZ 85287 USA; 6 Neuro-Oncology Research, Barrow Neurological Institute, St. Joseph’s Hospital and Medical Center, Phoenix, AZ USA; 7 Department of Neurosurgery, Neurosurgery Research Lab, Barrow Neurological Institute, St. Joseph’s Hospital and Medical Center, Phoenix, AZ USA; 8 BNI-ASU Center for Preclinical Imaging, Barrow Neurological Institute, St. Joseph’s Hospital and Medical Center, Phoenix, AZ USA

## Abstract

Five immunocompetent C57BL/6-cBrd/cBrd/Cr (albino C57BL/6) mice were injected with GL261-luc2 cells, a cell line sharing characteristics of human glioblastoma multiforme (GBM). The mice were imaged using magnetic resonance (MR) at five separate time points to characterize growth and development of the tumor. After 25 days, the final tumor volumes of the mice varied from 12 mm^3^ to 62 mm^3^, even though mice were inoculated from the same tumor cell line under carefully controlled conditions. We generated hypotheses to explore large variances in final tumor size and tested them with our simple reaction-diffusion model in both a 3-dimensional (3D) finite difference method and a 2-dimensional (2D) level set method. The parameters obtained from a best-fit procedure, designed to yield simulated tumors as close as possible to the observed ones, vary by an order of magnitude between the three mice analyzed in detail. These differences may reflect morphological and biological variability in tumor growth, as well as errors in the mathematical model, perhaps from an oversimplification of the tumor dynamics or nonidentifiability of parameters. Our results generate parameters that match other experimental *in vitro* and *in vivo* measurements. Additionally, we calculate wave speed, which matches with other rat and human measurements.

## Introduction

Glioblastoma multiforme (GBM) is an aggressive form of brain cancer with mean survival time following diagnosis ranging from about 4 months with supportive care only^1^ to 15 months with standard treatment^2, 3^. GBM cells have both proliferative and migratory characteristics. Although surgical resection may remove the bulk of the original tumor, it has long been recognized that GBM cells migrate away from the main mass and penetrate the brain parenchyma^4^, forming recurrent tumors adjacent to and/or extending from the previous surgical resection bed.

GBM has been the subject of many previous mathematical modeling attempts. Work by Frieboes, Lowengrub, and their collaborators^5–7^ captures the angiogenic and invasive behavior of growing gliomas at the microscale. At the macroscale, human GBM is characterized by a diffusive as well as invasive growth pattern, for which partial differential equation (PDE) models are used. One early effort used a reaction-diffusion model to capture the gross behavior of GBM tumors as well as the effects of treatment^8^. Alvord^9^ was able to estimate the recurrence of GBM after resection using similar models.

One popular reaction-diffusion model is

1 $$\frac{{\rm{\partial }}u}{{\rm{\partial }}t}(t,{\bf{x}})=\mathop{\underbrace{{\rm{\nabla }}\cdot (D{\rm{\nabla }}u(t,{\bf{x}}))}}\limits_{{\rm{d}}{\rm{i}}{\rm{f}}{\rm{f}}{\rm{u}}{\rm{s}}{\rm{i}}{\rm{o}}{\rm{n}}\,}+\mathop{\underbrace{\rho u(t,{\bf{x}})(1-u(t,{\bf{x}}))}}\limits_{{\rm{g}}{\rm{r}}{\rm{o}}{\rm{w}}{\rm{t}}{\rm{h}}\,},$$

where *u* expresses a normalized tumor cell density (0 ≤ *u*(*t*, **x**) ≤ 1) at a particular time *t* and brain location **x**. The parameter *D* governs the rate of diffusion, and *ρ*, the growth rate of the tumor cell population. Swanson and her collaborators have explored the potential utility of equation (1) as a “patient specific” mathematical model to estimate patient survival^10^ and to optimize treatment strategies^11^. They have attempted to estimate the parameter *ρ* and the ratio *ρ*/*D* from successive magnetic resonance (MR) images of individual patients: small values of the latter ratio appear to correspond to especially “diffuse” growth patterns, and larger values to more “nodular” tumors^11^. Such characterizations may predict which patients are likely to derive the greatest benefit from surgical resection^12^.

The mathematical modeling of human GBM is an inherently challenging enterprise. GBM may arise *de novo* or as a malignant progression from a preexisting lower-grade lesion^13^, and the extent of GBM invasion away from the main mass is difficult to ascertain from MR images^14^. Thus, the initial conditions in an individual patient case are uncertain. In addition, multiple genetic pathways are involved in the development and progression of GBM^13^. The expression of specific genes and gene pathways affect the rate at which GBM tumors proliferate and spread^11^. GBM invasion may also be facilitated by other cell types, including macrophages and microglia^15^. Thus, the ability to estimate growth and diffusion constants in any mathematical model of an individual tumor is limited. Finally, models like equation (1) do not explicitly account for immune response, genetic drift in the tumor, or the effects of treatment. Despite these shortcomings, equation (1) has the virtue of simplicity, particularly given the limited frequency of clinical imaging data and the uncertainties surrounding many of the biological details of GBM growth, progression, and treatment.

These considerations motivate the following controlled laboratory experiment. Using a well characterized murine glioma cell line (the details of which are provided in the methods section), we injected five mice intracranially to provide tumor initial conditions that were as similar as possible. The growth of each tumor was followed in five successive MR imaging sessions designed to yield high-resolution images. Our objective is to determine how well simple models like equation (1) can predict the future growth of each mouse’s tumor using high-quality MR imaging alone, based on various assumptions of the initial conditions, tumor cell densities, and model parameters, as estimated from the images. Our simulations are performed in two and in three dimensions. We also characterize the uncertainties inherent in the predictions and in estimates of the model parameters using our methodology.

The GL261 cell line has been chosen because its diffusive and invasive growth characteristics in immunocompetent mice resemble those of human GBM^16–18^ and because it has been well studied (more details are given in the methods section). We report on our modeling results using data from three of the mice, due to difficulties in processing the images for the other two (see the methods section).

We begin by describing our mathematical models and the associated computational setup. This is followed by our description of the 3D finite difference model and results. Explanations of the 2D level set method and its results are described next. We then discuss the results and our future directions. At the end of the manuscript, we detail the methods and materials needed for generating the experimental data.

## Mathematical Modeling

### Overview of laboratory data and modeling frameworks

**Figure 1:**
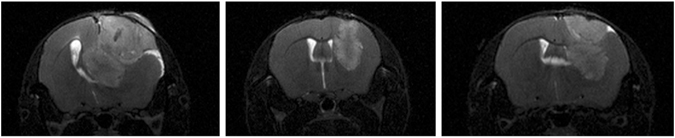
T2-weighted MR images taken 25 days after inoculation at the same location in the brain with respect to the initial needle track of the three mice used in this study. The left panel represents Mouse 1, the center, Mouse 2, and the right panel, Mouse 3. Despite inoculations with the same volume of tumor cells, the final tumors varied considerably in size.

Figure 1 shows a representative T2-weighted MR image of each mouse at the last imaging session, 25 days after implantation. T2-weighted imaging produces image contrast where regions of increased water content in the tumor appear bright in the image. These bright regions indicate edematous brain tissue surrounding the tumor where the blood-brain barrier is disrupted, and in which there are spreading tumor cells. This method allows visualization and volume measurement with the use of contrast agents and is commonly used in clinical settings to evaluate the extent and mass effect of gliomas. Although each mouse was inoculated with approximately the same number of cells from the same culture, the visible volumes of the final tumors range from approximately 12 mm^3^ to 62 mm^3^, with considerable differences in mass effect. Table 1 shows estimated visible tumor volumes in T2-weighted images for each mouse at each of the five imaging time points. The sizes of the tumors diverge considerably during the last ten days of growth. Although our experimental sample is too small to provide meaningful statistics, our results suggest that there is significant variability in the *in vivo* progression of GL261 tumors from similar intracranial inoculations.

**Table 1:** Volumes of visible tumor on MRI of each mouse at each time point.

Mouse	Day 11 volume (mm^3^)	Day 15 volume (mm^3^)	Day 18 volume (mm^3^)	Day 22 volume (mm^3^)	Day 25 volume (mm^3^)
1	0.715	1.155	4.980	15.570	25.460
2	0.595	3.025	4.320	8.095	12.340
3	0.970	3.855	16.485	30.675	61.490

The discussion in this section compares the ability of two mathematical modeling frameworks to reproduce the experimentally observed growth and diffusion of the laboratory tumors using high-resolution, T2-weighted MR images. We compare a 3-dimensional (3D) finite difference method and a 2-dimensional (2D) level set method in terms of accuracy, as measured by the degree of overlap via the Jaccard distance (defined below) between the simulated and observed tumors in the appropriate computational domain. Our efforts may provide insight into nature of the model errors that may arise in the more complicated case of human GBM.

Early mathematical modeling of GBM growth and diffusion were based on reaction-diffusion equations. Tracqui *et al*.^8^ formulated the earliest reaction-diffusion model applied to GBM and simulated the effect of treatment. Others expanded upon this basic model, incorporating a spatially-dependent diffusion coefficient that varied between gray and white matter tracts^19, 20^. These models are effective in determining the proliferating core of the tumor but are not as good at estimating the invasive front.

Later modeling efforts have incorporated a “go or grow” hypothesis, in which GBM tumor cell populations are assumed to consist of two phenotypes: a migratory subpopulation and a stationary, proliferative subpopulation^21, 22^. In contrast, a recent paper^23^ shows that a model with a single tumor cell population, coupled with a density-dependent diffusion term, suffices to produce similar growth dynamics. The mathematical models described here incorporate only a single tumor cell phenotype, as our focus is on the predictive accuracy of equation (1), given its previous application to human GBM cases.

Recent *in vivo* modeling efforts have incorporated anisotropic diffusion and make use of diffusion-weighted imaging (DWI), which uses local diffusion of water molecules to generate image contrast. From the 6 (or more) diffusion images, a diffusion tensor can be created. This method, known as diffusion tensor imaging (DTI), enables the indirect measurement of local diffusion orientation and the degree of local diffusion anisotropy^24^. It is unclear how to relate water diffusion from DTI directly to tumor cell diffusion, though several groups have proposed various methods^25–30^. Other authors have considered a spatially dependent diffusion coefficient, with different values for regions of gray and white matter^31^. However, since mouse brains have a relatively uneven distribution of gray and white matter as compared to humans, we consider only a diffusion constant that is uniform in space. For a recent review on GBM modeling efforts, please see Martirosyan *et al*.^32^.

The simulations presented below are done in both 2D and 3D. The 3D simulations, presented first, represent our best efforts to capture the observed tumor volume of each mouse under three different hypotheses regarding constant versus time-varying parameters. A limitation of the 3D approach is that we are unable to capture anisotropic effects of diffusion. We introduce a 2D model to examine how including anisotropic diffusion and time-dependent brain geometry alters our ability to reproduce the experimental data. Other researchers have had great success using 2D models^33^.

### Computational Setup

Our 3D simulations use a finite-difference approach, in which the brain domain Ω consists of the imaged brain tissue with the ventricles segmented out. The notation **x** = (*x*, *y*, *z*) refers to the spatial location within Ω. For the 2D level set simulations, the domain is a plane as seen in one slice of an MR image, and **x** = (*x*, *y*). The boundary conditions in both cases are no-flux (the tumor does not penetrate the skull or the ventricles):

2 $$\begin{array}{cc}\frac{\partial }{\partial n}u(t,{\bf{x}})=0, & {\bf{x}}\in \partial {\rm{\Omega }}\end{array}.$$

Several considerations affect our choices of the initial conditions. Although we know how many cells were implanted intracranially, their initial distribution is difficult to ascertain, for two reasons. First, there may have been backsplash during the implantation process. The needle creates a divot into the brain through which tumor cells may flow back towards the surface of the brain. Second, although we know the cell concentration and percentage of viable cells in the initial inoculum, there may be differences from mouse to mouse in how many of those cells survived the implantation and began to grow. For this reason, the simulations described below are started from initial conditions based on the first imaging session of the tumor (day 11 after implantation).

The cell density within the enhancing regions corresponding to tumor is uncertain. We have assumed that *u*(*t*_0_, **x**) = 0.5, i.e., half the maximum density, within regions corresponding to visible tumor for each simulation interval starting at *t*_0_. Although this choice of initial density is arbitrary, the computational results are not especially sensitive to its value (more details are provided below).

Simulations are run from day 11 to day 25. Synthetic T2-weighted MR images are created by assuming that tumor cell densities greater than 0.16 (the carrying capacity is 1) correspond to “visible tumor” on the MR scan^34^. We compare the visible tumor volume on the synthetic MR images with that of the actual T2W MR images using an error function based on the Jaccard distance at each imaging time point. The Jaccard distance is a measure of the distance between two sets *A* and *B* at time *t*, given by

3 $${d}_{J,t}(A,B)=1-\frac{|A\cap B|}{|A\cup B|}.$$

In this application, *A* is the set of voxels in the simulated MR image and *B* the set voxels corresponding to enhancing tumor. If *A* and *B* are disjoint, then *d*_*J*,*t*_(*A*, *B*) = 1, and if *A* = *B*, then *d*_*J*, *t*_(*A*, *B*) = 0.

Equation (1) contains two parameters: the proliferation rate *ρ* and the diffusion rate *D*. One natural question is whether, under suitable assumptions, these two parameters can be estimated from the MR data using the Jaccard distance as an error function. Furthermore, we *want* to quantify the uncertainties in the parameter estimates. The next section describes three different hypotheses regarding the time dependence of *ρ* and *D* and the results of a commonly used optimization algorithm to obtain a “best guess” of their values. One of our main results is that, although it is possible to find parameter values that provide a reasonably good fit to the observed MR images of each mouse, uncertainty quantification is problematic—and may have implications for clinical applications in human cases.

## 3D Finite Difference Model

As discussed in the methods section below, the computational domain Ω for each mouse is the linearly interpolated uniform grid. The uniform grids of the brain and ventricles are imported into MATLAB. Then the ventricles are segmented out of the brain geometry to produce the computational domain. Since the ventricles have been segmented out, they are ignored in the discretization and no diffusion or growth occurs in the ventricular space, consistent with the boundary conditions. As the tumor grows, it begins to displace the surrounding brain tissue. At each scan session, the ventricles of each mouse brain have shifted slightly from the previous session due to mass effect. The gross anatomy of the brain has been registered; however, the internal structures, including the ventricles, shift due to mass effect of the growing tumor. We fix the locations of the ventricles to correspond to last MR scan in our computational geometry.

As mentioned above, we obtained usable time sequences of images for three mice, which we call Mouse 1, 2, and 3 in the results presented below. It is not possible to segment out the ventricles for the first two time points (days 11 and 15) for Mouse 3; simulation results for this mouse are carried out only for a truncated time period. (It is possible, however, to estimate the total visible tumor volume in all three mice at each time point, so Table 1 gives a complete set of imaging volumes).

The model is discretized using centered finite differences in space. MATLAB’s ode45, a variable time-step, explicit Range-Kutta 4/5 solver, is a suitable integrator for this purpose. (Explicit schemes for finite difference methods are not always stable.) The space discretization is chosen to correspond to the pixel width in the postprocessed images. (The scans have a resolution of 0.1 mm × 0.1 mm × 0.5 mm). The full code is publicly available, as detailed at the end of the manuscript.

### Simulation Hypotheses

We investigate the values of *D* and *ρ* necessary to approximate the observed experimental tumor volumes. From the segmented tumors in the MR images, we calculate the volume of the visible tumor in MIMICS based on the number of pixels that are segmented as tumor (Table 1). The final tumor sizes on day 25 vary from approximately 12 mm^3^ to 62 mm^3^, so we explore several hypotheses that may explain this observed range of values.

- **Hypothesis 1**: *The natural variance in the growth rate ρ and diffusion rate D account for the large difference in final tumor size*. In the first set of simulation results described below, we initialize the model based on the first MR imaging on day 11 following implantation. Our goal is to determine how well an optimized choice of constant (averaged) parameter values fits the observed images on day 25 and how well they agree with values estimated elsewhere in the literature.
- **Hypothesis 2**: *The values of D and ρ are time dependent*, *corresponding to morphological changes during the growth of the tumors*. A small tumor may be highly proliferative, but as the tumor grows and resources become scarce, the growth dynamics may be dominated by cell migration away from the main mass. In the second set of simulation results below, we attempt to optimize the values of *D* and *ρ* from time point to time point and see whether all of the mice display similar trends in the variation of these parameters.
- **Hypothesis 3**: *In addition to the selection of optimized model parameters from one imaging time to the next*, *updates to the initial conditions at each imaging time point are made based on the visible tumor in each scan*. This “predictor-corrector” approach is analogous to that used in operational weather forecasting, where the initial conditions of the weather model are updated periodically in view of incoming observations.

**Figure 2:**
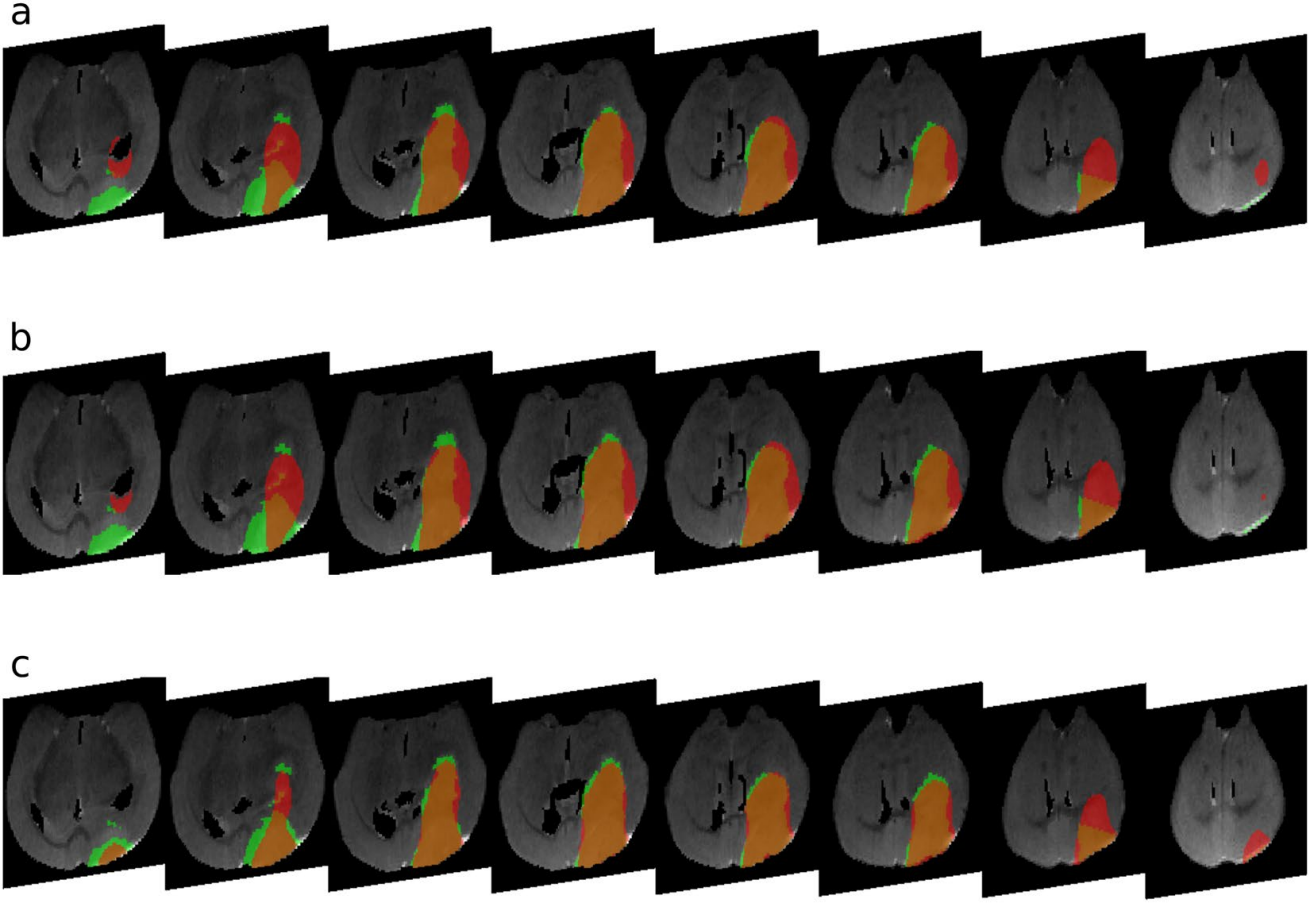
Representative simulation results for Mouse 1 at day 25 following implantation under (**a**) Hypothesis 1, (**b**) Hypothesis 2, and (**c**) Hypothesis 3. The simulated enhancing tumor volume is shown in red, and the segmented regions of enhancement in the laboratory tumor in green for eight different slices of the MR images.

We optimize the vector of model parameters *θ* = (*D*, *ρ*) for each mouse individually using the fminsearch command in MATLAB under each of the three hypotheses. The optimization process seeks to minimize an error function based on the time average of the sum of the Jaccard distances, given by

4 $$E(D,\rho )=\frac{1}{n}\sum _{k=1}^{n}{d}_{J}(A(D,\rho ,{t}_{k}),B({t}_{k}))$$

where *A*(*D*, *ρ*, *t*_*k*_) corresponds to the enhancing voxels at time *t*_*k*_ as predicted by model (1) for a given pair of parameters, and *B*(*t*_*k*_) corresponds to the voxels on the original MR image which are deemed to be enhancing at time *t*_*k*_. The summation is performed over all *n* time points for which imaging is available. For Mouse 1 and 2, there are *n* = 4 subsequent images at day 15, 18, 22, and 25 following implantation; for Mouse 3, there are *n* = 2 subsequent images at days 22 and 25. As an example, Fig. 2 shows *A* in red and *B* in green for each hypothesis for each of the available MR image slices for Mouse 1 at time point 5, *t*_*k*_ = 25 days after implantation.

Equation (4) measures only the extent of overlap of the enhancing region of the tumor. The actual tumor boundaries are larger, but the extent to which the glioma cells infiltrate the brain beyond the visible tumor margin in the T2-weighted images is unknown. Konukoglu *et al*.^28^ have suggested an estimation procedure based on equation (1) to extrapolate the tumor cell density distribution beyond the visible margin in human GBM. Since we do not have histological data with which to compare, we measure only the enhancing volumes (“visible tumor”) for the murine gliomas. For the purposes of the simulations reported here, we assume that the detection threshold corresponds to *u* = 0.16^34^, but there is considerable uncertainty in this value. This question can be resolved only by correlating histology slides from the mouse brains with MR final images, a project that will be undertaken in future work.

### Simulation Results

We present three sets of simulation results, each with a different hypothesis regarding the time evolution of the parameters *D* and *ρ* in model (1).

#### Hypothesis 1

Hypothesis 1 assumes that the tumor grows according to fixed values of the parameters *D* and *ρ*, but that these parameters may differ from mouse to mouse. We select initial conditions corresponding to the visible tumor at the first MR imaging session (day 11 following implantation). We use MATLAB’s fminsearch function to determine values of *D* and *ρ* that minimize the Jaccord distance error, equation (4), over the subsequent four imaging time points at days 15, 18, 22, and 25 following implantation. The resulting values, shown in Table 2, range from $\rho =0.0167\,\,{{\rm{h}}}^{-1}\,{\rm{to}}\,\rho =0.0188\,{{\rm{h}}}^{-1}$ and from $D=319.22\,\mu {{\rm{m}}}^{2}/{\rm{h}}$ (Mouse 2) to $D=651.17\,\mu {{\rm{m}}}^{2}/{\rm{h}}$ (Mouse 3).

**Table 2:** Values of estimated parameters for Mouse 1, 2, and 3 and each hypothesis at each time point using the 3D finite difference method.

Mouse	Hypothesis	Time Point	*D* (*μ*m^2^/h)	*ρ* (h^−1^)	velocity $2\sqrt{D\rho }$(*μ*m/h)	Error
1	1	—	413.77	0.0188	5.5781	0.4524
	2	2	139.24	0.0182	3.1838	0.1196
		3	839.93	0.0248	9.1280	0.1191
		4	1047.6	0.0192	8.9697	0.1029
		5	968.75	0.0082	5.6369	0.0949
	3	2	139.24	0.0182	3.1838	0.1196
		3	233.97	0.0499	6.8338	0.1145
		4	1156.2	0.0178	9.0731	0.0688
		5	1305.6	0.0105	7.4051	0.0644
2	1	—	319.22	0.0167	4.6178	0.4528
	2	2	558.74	0.0235	7.2472	0.1151
		3	206.21	0.0100	2.8720	0.1067
		4	346.35	0.0055	2.7604	0.1042
		5	886.07	0.0104	6.0713	0.0979
	3	2	558.74	0.0235	7.2472	0.1151
		3	950.79	0.0051	4.4041	0.0846
		4	77.734	0.0369	3.3873	0.0621
		5	94.161	0.0520	4.4255	0.0643
3	1	—	651.17	0.0177	6.7899	0.2833
	2	4	859.70	0.0127	6.6085	0.1408
		5	454.29	0.0236	6.5487	0.1364
	3	4	859.704	0.0127	6.6085	0.1408
		5	1552.1	0.0200	11.1431	0.1027

Minimization is performed with respect to equation (4).

Figure 2 part (a) superimposes the simulated tumor in red, and the experimentally segmented tumor in green, over representative MR images at the last imaging time point for Mouse 1. The Jaccard distance error is $E(D,\rho )=0.4524$. The full time point fits for Mouse 1 (Supplementary Fig. S1), Mouse 2 (Supplementary Fig. S4) and Mouse 3 (Supplemenary Fig. S7), are shown in the Supplementary Figures.

#### Hypothesis 2

Hypothesis 2 asserts that there may be a morphological change that occurs sometime within the tumor growth process that changes the values of *D* and *ρ*. The tumor grows more quickly when it is small, but as time proceeds and resources become scarce, the growth rate may slow.

The parameter estimation procedure under this hypothesis proceeds in a similar way to that in Hypothesis 1, except that the parameter optimizations are done between successive imaging time points. That is, we take the same initial conditions on Day 11, then run fminsearch to minimize *E*(*D*, *ρ*) at day 15 only. Then, starting with the simulated tumor at day 15, we select parameter values to minimize *E*(*D*, *ρ*) at Day 18, and so on. Table 2 shows all the results, which for Mouse 1 gives the estimates $\rho =0.0182,0.0248,0.0192,0.0082\,{{\rm{h}}}^{-1}$, and $D=139.24,839.93,1047.6,968.75\,\mu {{\rm{m}}}^{2}/{\rm{h}}$ for the Day 11–15, 15–18, 18–21, and 21–25 time intervals, respectively. The sum of the individual Jaccard error measures, equation (4), is 0.4365, representing a 3.5% decrease in Jaccard distance from Hypothesis 1. Figure 2 part (b) shows the results for Mouse 1 for day 25, the last time point. The results suggest that there is a significant temporal variation in the model parameters as the tumor evolves. However, the model’s ability to track the visible tumor is only slightly improved compared to Hypothesis 1. The fit for all time points for Hypothesis 2 for Mouse 1 (Supplementary Fig. S2), Mouse 2 (Supplementary Fig. S5) and Mouse 3 (Supplementary Fig. S8) are available in the Supplementary Figures.

#### Hypothesis 3

Hypothesis 3 also asserts that *D* and *ρ* to vary with time, but the model is restarted from new initial conditions each time. That is, we optimize the parameters from time point to time point as in Hypothesis 2, but in addition, we reset the initial conditions at each imaging time point, under the assumption that tumor cell densities at and above 0.16 correspond to visible tumor on T2-weighted images. The optimized estimates for Mouse 1 for the growth rate are $\rho =0.0182,0.0499,0.0178,0.0105\,{{\rm{h}}}^{-1}$ and $D=\mathrm{139.24,233.97,1156.2,1305.6}\,\mu {{\rm{m}}}^{2}/{\rm{h}}$ for the Day 11–15, 15–18, 18–21, and 21–25 time intervals, respectively. The sum of the individual Jaccard error measures (4), based on the predicted images prior to the initial condition updates, is 0.3663, representing a decrease of 18.8% from Hypothesis 1. Figure 2 part (c) shows the results for Mouse 1 at the last time point, day 25. For full results of Hypothesis 3, see the supplementary figures for Mouse 1 (Supplementary Fig. S3), Mouse 2 (Supplementary Fig. S6), and Mouse 3 (Supplementary Fig. S9).

For two of the three mice, the diffusion coefficient appears to increase over time. However, for Mouse 2, the optimized values for *D* decrease significantly over the last two time points, but the growth parameter *ρ* increases sharply. This result may reflect a biological difference in the growth dynamics of Mouse 2’s tumor compared to the others.

#### Velocity of Tumor Boundary

Since our analysis of Hypotheses 1–3 in the previous sections indicates that assuming constant *D* and *ρ* is inadequate to accurately model tumor growth and diffusion, we examine the dependence of the velocity of the visible tumor boundary on the volume to determine if the estimated parameters correlate with the volume of the tumor.

**Figure 3:**
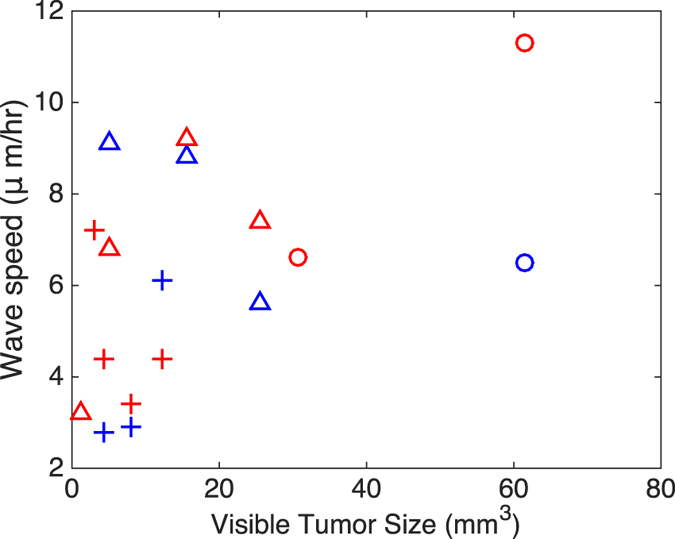
Dependence of wave speed of tumor on tumor size. The wave speed (velocity) of the tumor is calculated by $2\sqrt{D\rho }$. Blue markers show results from parameters optimized under Hypothesis 2; red markers, Hypothesis 3. Triangles: Mouse 1; pluses: Mouse 2; circles: Mouse 3.

Model (1) implies that the tumor boundary moves as a traveling wave whose wave speed (velocity) is $2\sqrt{D\rho }$. Figure 3 shows a plot of the predicted wave speed versus the visible tumor boundaries under each set of modeling hypotheses. The data points are separated by shape and color: blue markers correspond to the wave speeds calculated for Hypothesis 2 and red markers correspond to the wave speeds generated from Hypothesis 3. Within these colors, the markers signify the separate mice: Mouse 1 is represented by triangles, Mouse 2 with plusses, and Mouse 3 with circles. At first glance, it may appear that there is little correlation between the two variables. However, if we examine the Hypothesis-3 data (red markers), we can see there does appear to be some relationship between wave speed and visible tumor size. More data are necessary to quantify the relationship, however.

### Identifiability of Parameters

The minimization procedure described above yields estimates of the parameters *D* and *ρ* for equation (1) that are reasonably consistent with the experimental data. However, the procedure does not provide confidence intervals for the parameters. We turn to this question now.

Let *u*(*t*, *θ*) denote the output of a mathematical model at time *t* given a vector of parameters *θ*, whose components may include initial conditions, rate coefficients, and other model-related constants. Following Raue *et al*.^35^, one common approach to measuring the goodness of fit is to define a residual function of the form

5 $${\chi }^{2}(\theta )=\sum _{i=1}^{n}\,\sum _{k=1}^{p}\,{(\frac{{y}_{k}({t}_{i})-{u}_{k}({t}_{i},\theta )}{{\sigma }_{ki}})}^{2},$$

where ${u}_{k}({t}_{i},\theta )$ is the model prediction of the *k* th observable at time *t*_*i*_, *y*_*k*_(*t*_*i*_) is the corresponding measurement, and *σ*_*ki*_ is the standard error in *y*_*k*_(*t*_*i*_). The parameter estimate is $\widehat{\theta }$ = argmin*χ* ^2^(*θ*), which may be regarded as the minimizer of a likelihood function when the measurement noise is unbiased and gaussian.

The parameter vector *θ* is *identifiable* if $\hat{\theta }$ is unique for a given set of measurements and associated uncertainties. The components of *θ* may be *nonidentifiable* if one or more of them is an implicit function of the others, or if the set of observables is insufficient to find a unique minimizer of equation (5). In addition, *θ* may be *practically nonidentifiable* ^35^ if the uncertainties *σ*_*ki*_ are large enough to make it difficult to distinguish between possible minimizers, i.e., the value of *χ* ^2^(*θ*) changes little for a large range of *θ*.

In our case, it is difficult to define a sum of squares like equation (5), because we do not know the correlation between the tumor cell density and the degree of MR enhancement within each voxel, and so we cannot define an associated standard error. Instead, our simulation and measurement thresholds are binary: a given grid point in the computational domain contains tumor cells above a detectable threshold or it does not, and each pixel of the corresponding MR image either shows enhancement or it does not.

**Figure 4:**
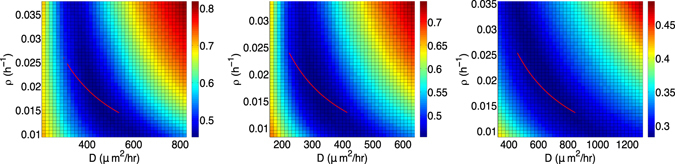
Plots of *E*(*D*, *ρ*) (equation (4)) for Hypothesis 1 for Mouse 1 (left), Mouse 2 (center), and Mouse 3 (right) for selected intervals of the model parameters *D* and *ρ*. The red line represents the wave speed, $2\sqrt{D\rho }$, for the “optimized” values of *D* and *ρ*.

Figure 4 shows a plot, for each of the three mice, of *E*(*D*, *ρ*) (equation (4)) for *D* and *ρ* in the indicated intervals. The values of *E*(*D*, *ρ*) are color-coded from deep blue (smaller values, corresponding to smaller Jaccard distances between predicted and observed regions of visible tumor) to deep red (larger values, corresponding to larger Jaccard distances). In each case, there is a deep blue “valley” of minimal values of *E*(*D*, *ρ*), which suggests that the parameters *ρ* and *D* in equation (1) are nonidentifiable: many choices of the parameters are consistent with the available data. Consequently, it is not possible to formulate confidence intervals around the values of *D* and *ρ* obtained from the error minimization procedure described above.

Model (1) implies that the tumor front expands like a traveling wave with wave speed $c=2\sqrt{D\rho }$. The “optimal” value of *c* for each mouse is derived from the minimization procedure in the previous section and appears in the sixth column of Table 2. Under Hypothesis 1, *c* is constant throughout the simulation. Superimposed on each plot in Fig. 4 is a red arc showing the values of *D* and *ρ* corresponding to the wave speed *c* obtained from the corresponding mouse (i.e., $\rho (D)={c}^{2}/4D$) for a selected interval of *D*. The results for Mouse 3 suggest that *c* may be identifiable in some cases, as the arc corresponds approximately to the smallest values of *E*(*D*, *ρ*) in the indicated range.

Although the parameters *ρ* and *D* for our tumor growth model cannot be estimated uniquely using the available imaging data, they may be possible to estimate from additional types of measurements, such as tumor cell counts from cell histology. Such measurements may define a quantitative relationship between the degree of image enhancement and tumor cell density. Such efforts will be the subject of future research.

### Initial Condition Sensitivity Analysis

We have performed additional analyses to determine the sensitivity of the parameter estimation procedure to the choice of initial condition. Using Mouse 1, we have repeated the parameter estimation procedure assuming that the voxels corresponding to visible tumor are at 0.3 and 0.7 of the carrying capacity. The results, shown in Table 3, are rather insensitive to the initial starting densities. Although the estimated values of *D* increase with the initial cell density, the values of *ρ* decrease: a high initial density of cells supports more diffusion and less growth. Nevertheless, the predicted wave speeds given by $2\sqrt{D\rho }$, are all approximately the same.

**Table 3:** Values of estimated parameters for Mouse 1 using initial condition cell densities for Hypothesis 1.

Initial Condition Cell Density (%)	*D* (*μ*m^2^/h)	*ρ* (h^−1^)	velocity $2\sqrt{D\rho }$ (*μ*m/h)	Error
30	316.43	0.0226	5.3484	0.4510
50	413.77	0.0188	5.5781	0.4524
70	441.52	0.0168	5.4476	0.4592

Minimization is performed with respect to equation (4).

## 2D Level Set Method Model

### Modifications to Model Equations

This section describes a reformulation of the model (1) as a two-dimensional free boundary problem, also known as a Stefan problem. Although our data are three dimensional, we examine two-dimensional coronal, sagittal, and transverse (horizontal) sections to explore the effects of anisotropy and time-dependent brain geometry on the growth of the simulated tumor and to determine whether a two-dimensional model can capture projections of the three-dimensional movement. The Stefan problem was first derived for the transfer of heat during solidification or melting processes where the boundary is the phase boundary separating regions of different temperatures. More recently, free boundary problems have been studied in the context of wound healing and tumor growth^33, 36–40^.

**Figure 5:**
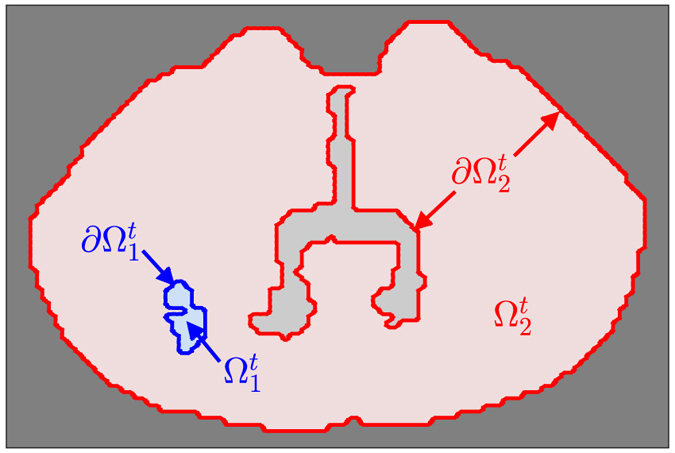
Schematic illustration of the computational domain of the moving boundary initial value problem for tumor growth. The blue area (${{\rm{\Omega }}}_{1}^{t}$) represents the tumor cells, the light gray area represents the ventricles, the red area (${{\rm{\Omega }}}_{2}^{t}$) represents the remainder of the brain, and the dark gray represents the area outside of the brain.

We assume the governing equation for brain tumor growth is equation (1), where the tumor domain, denoted ${{\rm{\Omega }}}_{1}^{t}$, is time dependent; ${{\rm{\Omega }}}_{1}^{0}$ consists of those locations where tumor is visible on the initial scan at day 11. The remainder of the brain is denoted ${{\rm{\Omega }}}_{2}^{t}$; see Fig. 5.

Similar to the three-dimensional simulations discussed previously, the initial condition is

$$\begin{array}{cc}u(0,{\bf{x}})=0.5, & {\bf{x}}\in {{\rm{\Omega }}}_{1}^{0}\end{array},$$

in interior regions of the enhancing tumor, and

$$\begin{array}{cc}u(0,{\bf{x}})=0.16, & {\bf{x}}\in \partial {{\rm{\Omega }}}_{1}^{0}\end{array},$$

on the boundary, corresponding to the hypothesized detection threshold *u* = 0.16^34^ from T2-weighted images. The no-flux boundary condition is

6 $$\nabla u\cdot {{\bf{n}}}_{2}=\mathrm{0,}\,\,{\rm{on}}\,\partial {{\rm{\Omega }}}_{2}^{t},$$

where **n**_2_ is the unit normal to the boundary ${{\rm{\Omega }}}_{2}^{t}$.

We assume that the velocity of the tumor boundary **v**(*t*, **x**) is governed by Darcy’s law, ${\bf{v}}=-\,\mu \nabla p$, where *μ* may be interpreted as the cellular mobility, assumed equal to *D* to avoid an extra model parameter. The tumor-generated pressure *ρ* is modeled by $p(\rho )=\,\mathrm{ln}(\rho )$. This choice of pressure allows for an arbitrarily large stress to stretch and compress of the tumor tissue. These assumptions lead to the Stefan condition

7 $${\bf{v}}\cdot {{\bf{n}}}_{1}=-(\frac{D}{\bar{u}}){\rm{\nabla }}u\cdot {{\bf{n}}}_{1},\,\,{\rm{o}}{\rm{n}}\,{\rm{\partial }}{{\rm{\Omega }}}_{1}^{t},$$

where $\bar{u}=0.16$ and **n**_1_ is the unit normal to the boundary ${{\rm{\Omega }}}_{1}^{t}$. The calculation of the velocity of the boundary of the visible tumor here depends on equation (7), not on the value $2\sqrt{D\rho }$ as in the case of the 3D finite difference model. Because the ventricles are displaced while the tumor moves, the geometry of ${{\rm{\Omega }}}_{2}^{t}$ is updated in time on days 15, 18, 22, and 25 directly from the corresponding MR scans.

A numerical solution of the Stefan problem for a given tumor and parameters *D* and *ρ* is computed using a level set method introduced by Osher and Sethian^41^ and applied to Stefan problems by Chen *et al*.^42^, Javierre *et al*.^43^, and Arciero *et al*.^44^, among others. Supplementary Methods S3 contains full details of the solution method.

## Results

To fit the mathematical model in the previous section to experimental data of tumor growth in 2D sections of mice brains, we estimate the model parameters *D* and *ρ* by minimizing the Jaccard distance error, equation (3), and the mean-squared difference between the boundary points of the numerical simulation and the experimental data,

$${z}_{d}=\sum _{t=2}^{5}\,{(\sum _{h=1}^{L}\frac{{d}_{min,h,t}^{2}}{L})}^{1/2}$$

where the minimum distance *d*_min, *h*, *t*_ is calculated by minimizing the distance between every point *h* on the numerical boundary to every line segment along the data boundary at each imaging time point *t* (days 15, 18, 22, and 25). The boundary of the simulated tumor is easier to define than in the three-dimensional simulations, but the extent to which tumor cells have diffused beyond the visible edge is unknown. The weighted total error is given by

8 $${E}_{2}(D,\rho )={z}_{d}+10\sum _{t=2}^{5}{d}_{J,t}(A,B),$$

which is minimized as a function of the model parameters *D* and *ρ* using the MATLAB command fminsearch. The factor of 10 is included so that the error term from the boundary positions is of the same order as the Jaccard distance.

**Figure 6:**
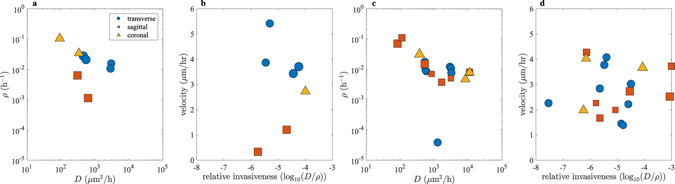
Parameter values for Mouse 1 and 2 from the 2D level set method. Estimated parameter values for various 2D slices as reported in Table 4: (**a**) Mouse 1 and (**c**) Mouse 2. The parameter estimation routine was run for various initial parameter value guesses, and the size of the data points corresponds to the magnitude of the error in equation (8). Radial velocity of the tumor taken from the last simulated day of movement versus relative invasiveness: (**b**) Mouse 1 and (**d**) Mouse 2.

We have fitted the experimental and numerical tumor edge positions by choosing *D* and *ρ* to minimize *E*_2_ for Mouse 1 using 4 transverse, 2 sagittal, and 2 coronal sections and for Mouse 2 using 8 transverse, 7 sagittal, and 3 coronal sections. These are all the sections that contain contrast-enhancing tumor over all time points. This analysis corresponds to Hypothesis 1 from the 3D finite difference simulations. Furthermore, the slices for which we can estimate parameters using the 2D level set method depend on whether the tumor has spread predominately radially in that section. Due to the software difficulties in obtaining ventricle data as previously described, we did not perform this analysis on Mouse 3. The estimated parameters are plotted in Fig. 6(a,c). The velocity of the tumor front as a function of the relative invasiveness, defined as ${\mathrm{log}}_{10}(D/\rho )$ ^12^, is plotted in Fig. 6(b,d). Table 4 displays all the computed values for *D*, *ρ*, and tumor velocity. The values of *D* and *ρ* appear to be independent of the choice of coronal, axial, or sagittal section, which suggests that there is no preferential direction for tumor growth. However, because the estimated parameter values vary throughout the brain, the results also imply that the tumor growth patterns are heterogeneous and cannot be well-modeled using constant values for the parameters *D* and *ρ*.

**Table 4:** Values of estimated parameters for Mouse 1 and 2 and Hypothesis 1 using the 2D level set method.

Mouse	Section	Slice	*D* (*μ*m^2^/h)	*ρ* (h^−1^)	velocity (*μ*m/h)	Error
1	Transverse	1	463.09	0.027164	3.6967	103.23
		2	554.98	0.02052	3.425	99.92
		3	3074.8	0.015377	5.4098	80.24
		4	2923.1	0.010395	3.8589	82.02
	Sagittal	1	305.51	0.0063098	1.2091	127.85
		2	626.77	0.0011284	0.3342	119.51
	Coronal	1	91.762	0.10424	2.8026	94.81
		2	336.48	0.034115	2.7293	91.14
2	Transverse	1	534.97	0.017231	3.0134	75.99
		2	2955.1	0.011975	4.0672	71.22
		3	3171.1	0.01068	3.7663	74.35
		4	3261.4	0.0074994	2.8306	73.75
		5	598.27	0.0084187	1.4412	67.44
		6	558.54	0.009343	1.3758	64.72
		7	1284.4	0.000037942	2.2519	71.19
		8	530.47	0.013664	2.21	68.79
	Sagittal	1	10984	0.0080234	4.2752	91.78
		2	1656.4	0.0037629	1.6703	87.20
		3	837.34	0.0070626	1.9906	73.21
		4	3098.5	0.0050553	2.2666	78.46
		5	83.08	0.070895	2.5196	107.28
		6	525	0.015	2.7293	115.82
		7	111.77	0.11002	3.7248	105.85
	Coronal	1	8317.9	0.004686	1.9781	84.79
		2	11120	0.007827	4.0281	79.72
		3	370.53	0.031518	3.6641	97.47

Minimization is performed with respect to equation (8).

**Figure 7:**
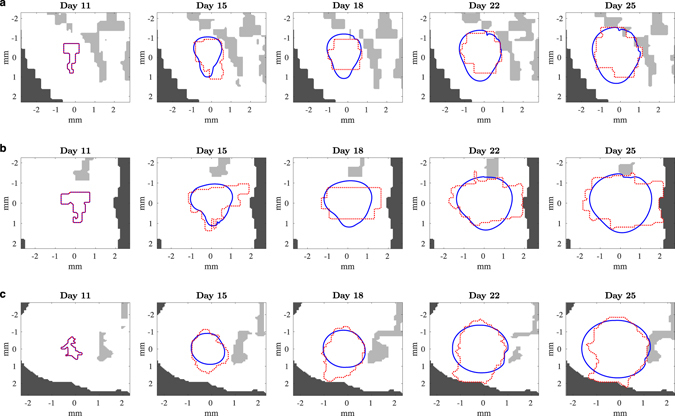
Simulation of 2D level set method for Mouse 2 with isotropic diffusion. Tumor boundary movement in Mouse 2 in select slices with estimated parameters from Table 4. The red dotted curve represents the tumor boundary from the laboratory imaging and the solid blue curve, the boundary from the 2D mathematical model. The light gray regions are ventricles and the dark gray regions are outside of the brain. (**a**) Transverse section; (**b**) sagittal section; (**c**) coronal section.

The intrinsic heterogeneity of the tumor growth pattern becomes apparent in the numerical simulations given in Fig. 7. Under the assumption of isotropic media, as described in the previous section, the level set method produces simulated movement that is symmetric and, in general, has a limiting elliptical shape, as shown by the solid blue curves in Fig. 7. The numerical simulations match the experimental tumors, shown by the dotted red curves, only if the observed tumor does not show a preferential direction for growth.

**Figure 8:**
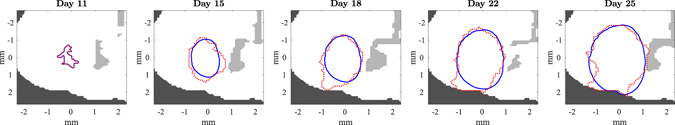
Simulation of 2D level set method for Mouse 2 with anisotropic diffusion. Tumor boundary movement in Mouse 2 in the same slice as Fig. 7(c) but with a spatially dependent value of the diffusion parameter *D*. Solid blue curve: numerical simulation; dotted red curve: actual tumor boundary. The light gray regions are ventricles and the dark gray regions are outside of the brain.

A contrasting simulation, in which the diffusion constant *D* varies spatially, is shown in Fig. 8. At each voxel, *D* is chosen randomly from a uniform distribution. In the horizontal direction on the left half of the computational domain for the brain section (as in Fig. 8), *D* is in the interval [0,0.2*η*], where $\eta =11120\,\mu {{\rm{m}}}^{2}/{\rm{h}}$, the same value of *D* given in Table 4 for Mouse 2, coronal slice 2. In the horizontal direction on the right half of the brain section, *D* is in [0,0.05*η*]. In the vertical direction, on the top half *D* is in [0.1*η*, 0.4*η*], and on the bottom half *D* is in [0.3*η*, 0.5*η*]. The average error *E*_2_ from equation (8) for 15 simulations is 70.66, which is smaller for this anisotropic case compared to the isotropic case given in Fig. 7(c). Although the error contribution from the Jaccard distance is not much different, the error contribution from the difference in boundary positions is smaller in the anisotropic case. The shapes of the tumor boundaries arising from the anisotropic assumption are a better match to the experimental data than the 3D simulations.

As with the 3D finite-difference method, we would like to determine whether the parameters are identifiable using our error function given by equation (8). Following the methods outlined in the previous section, Identifiability of Parameters, we created surface likelihoods for a subset of the 2D slices and parameter values. It appears that *D* and *ρ* remain non-identifiable for certain intervals of parameters.

## Discussion and Conclusions

In this study, we examine a laboratory murine model of GBM tumor growth, in which genetically similar, immunocompetent mice received identical intracranial injections from the same culture of a GL261-luc2 tumor line. The growth patterns of the resulting tumors vary considerably. From experimental MR images, we investigate two mathematical models and examine their ability to simulate the laboratory results under various choices of model parameters. The reaction-diffusion model, equation (1), is numerically integrated using a finite difference method in three dimensions. In two dimensions, an extension of equation (1) that incorporates a free boundary and an anisotropic domain is numerically integrated using a level set method.

Previous estimates of the growth parameter *ρ* is approximately in the range 0.014–0.018 h^−1^ for *in vitro* GL261-luc2 cells^45^ and 0.0096–0.011 h^−1^ for *in vivo* GL261-luc2 cells^46^. The estimated values for *ρ* in both sets of our simulations, presented in Tables 2 and 4, is comparable to these values.

Measurements of the apparent diffusion constant (ADC) for *in vivo* GL261-luc2 cells, which is calculated from diffusion weighted imaging that maps the diffusion of water molecules, range from 2.2–3.3 mm^2^/h^47, 48^. These values are orders of magnitude larger than the diffusion constant *D* estimated from our simulations in Tables 2 and 4. A more meaningful comparison between the two types of measurements requires a quantitative correlation between the diffusion of water in DWI images and the growth of the tumor over time, which likely will require cell density measurements from histology. Our largest value of *D* is still considerably less than previously published values, which suggests that errors in the initial conditions and in the assumptions on the correspondence between tumor cell densities and enhancing regions on MR images are contributing factors. Our assumption regarding the minimum cell density that is visible on MR may need to be revised significantly. The result of nonidentifiability of parameter estimations supports that more work must be done to quantify this relationship. Additionally, since parameter identifiability may depend on the initial condition of the system, revising our estimations of initial tumor cell density on enhancing regions of MR images on day 11 may alter the parameter identifiability results.

The 3D finite difference method applied to equation (1) implies that the velocity of the visible tumor boundary is $2\sqrt{D\rho }$, while the tumor boundary in a 2D level set method is estimated from the Stefan condition, equation (7). Converting to units of centimeters per year from Table 2, the 3D finite difference method yields velocity estimates from 2.789–7.996 cm/yr for Mouse 1, 2.516–6.348 cm/yr for Mouse 2, and 5.737–9.761 cm/yr for Mouse 3. In contrast, the 2D level set method produces the velocity estimates from 0.213–3.441 cm/yr for Mouse 1 and 0.875–2.719 cm/yr for Mouse 2 (see Table 4). The latter estimates are closer to those obtained from tumors in rat brains with implanted human glioblastoma cells^49^ and are on the low end of the human velocity range^50, 51^.

Within the 3D finite difference method, we formulated three hypotheses as to what contributed to the growth of the glioma in the murine brain. We noticed improvements, as measured by the Jaccard distance, from Hypothesis 2 (time-varying parameters) over Hypothesis 1 (constant parameters) of, on average, 4% (range 2.15– 6.38%). Similarly, we noticed improvements from Hypothesis 3 (short-term simulations) over Hypothesis 1 of, on average, 20.3% (range 14.05–27.98%).

From the preceding work, we draw the following conclusions.

- T2-weighted imaging alone is insufficient to uniquely estimate the model parameters in equation (1). Additional types of measurements are necessary to formulate confidence intervals on model parameters and associated predictions.
- Cancer cell densities derived from histology (stained microscope slices of the mouse brains), correlated with the MR imaging, may provide much more precise information about tumor cell distributions with which to evaluate the accuracy of associated mathematical models. These correlational data may permit a statistical characterization between tumor cell density and MR intensity enhancement that can make MR imaging data more useful for estimating initial conditions and identifying the model parameters. Finally, histological data may permit the formulation of more biologically realistic models.
- By performing simulations in two and three dimensions, we find comparable estimates of the model parameters *D* and *ρ* from the experimental data. This result suggests that the details of the time-varying geometry and mass effect omitted from our 3D simulations do not significantly affect the estimates of the model parameters. (A future laboratory effort involving more mice, as well as a 3D model incorporating mass effect, are needed to confirm this assertion).
- As a model for glioma growth, model (1) requires diffusion and proliferation parameters that vary with time. At the least, simulations with piecewise constant parameters maximize the overlap, measured by Jaccard distance, between the predicted and observed tumors. In particular, the simulation results under Hypothesis 3 suggest that incorporating periodic updates of initial conditions from MR imaging eventually may be a feasible way to make useful short-term predictions of the evolution of individual tumors. Accurate long-term predictions of tumor growth do not appear to be possible using our methodology.
- Model (1) is the simplest PDE to describe the dynamics of a diffusive, logistic growth process. Our results suggest that a somewhat more sophisticated model should include anisotropic diffusion. A density-dependent diffusion term may also be appropriate^23^.

If, after a careful histological examination of tumors from a future experimental effort involving more mice, it proves possible to determine a statistical correspondence between the local tumor cell density and MR imaging, then more sophisticated data assimilation methods can be applied to provide short-term forecasts of the evolution of the tumor, including predictions of the distribution of tumor cells in regions beyond the visible tumor boundary^52^. Finally, future research that elucidates additional details of glioma biology can inform potential improvements to model (1) to account for interactions with the immune system, genetic drift within the tumor cells, and other factors.

## Methods

### Animals and Care

The study follows the guidelines and regulations set by the National Institutes of Health Guide for the Care and Use of Laboratory Animals^53^ and approved by the Institutional Animal Care and Use Committee at the Barrow Neurological Institute and St. Joseph’s Hospital and Medical Center. All aspects of the experiments including animal housing, surgeries, imaging, and euthanasia were carried out at the Barrow Neurological Institute at St. Joseph’s Hospital and Medical Center. The animals had continuous free access to food and water.

### GL261-Luc2 Tumor Cell Line

The GL261-luc2/C57BL/6-cBrd/cBrd/Cr (albino C57BL/6) tumor model has been chosen because it is an established glioma model that is recommended for preclinical GBM therapy studies^54^. GL261-luc2 is a syngeneic tumor cell line originally developed within the immunocompetent C57BL/6J mouse strain^16, 54–57^. Seligman and Shear originally promoted the GL261 tumor cell line in the C57BL/6J mouse by implanting 3-methylcholanthrene pellets into the brains of mice in the 1930’s^16, 54, 55^. The GL261 cell line within the immunocompetent C57BL/6J mouse strain thus produces a more typical immune response to the glioma and is thought to involve a pathological progression of malignant glioma akin to that in humans. The GL261 cell line is an aggressive tumor that shares many characteristics similar to the human GBM: tumor necrosis, hemorrhages, angiogenesis, cellular proliferation, invasion, and inflammation^16–18^. Although GL261 has a tumoral cell rim, it is not the typical pseudopalisading architecture seen in human GBM^17^. However, gross cellular proliferation and invasion are the most important biological characteristics for this study in the context of modeling cellular growth and migration.

GL261 cells were obtained from the DCTD Tumor Repository (NCI, Frederick, MD) and grown in Dulbecco’s modified Eagle’s medium (DMEM) supplemented with 10% fetal calf serum (FCS) at 37 °C with 5% CO_2_. To facilitate a quantitative measurement of tumor growth rate, GL261 cells were stably transfected with the gene encoding Luc2 using the pGL4.51[luc2/CMV/Neo] vector (Promega Corp, Madison, WI) and FuGENE® 6 Transfection Reagent (Roche Applied Science, Indianapolis, IN) following conditions specified by the manufacturer. Stable transfectants were selected and maintained in DMEM containing 10% FCS and 100 *μ*g/ml Geneticin® (G418, Invitrogen Corp, Carlsbad, CA). These cells were designated GL261-Luc2; their growth rate is equivalent to the parental GL261 cell line^16, 58^.

### Implantation

GL261-Luc2 cells were harvested by trypsinization, washed and resuspended at a concentration of 1–2 × 10^7^ cells/ml in DMEM without FCS and implanted into ten-week-old immunocompetent C57BL/6-cBrd/cBrd/Cr (albino C57BL/6) mice (The Jackson Laboratory, Bar Harbor, ME, USA) with an average weight of 20 g. Briefly, the animals were anesthetized by an intraperitoneal injection of ketamine (10 mg/kg) and xylazine (80 mg/kg), placed in a stereotactic apparatus, and an incision was made over the cranial midline. A burrhole was made 0.1 mm posterior to the bregma and 2.3 mm to the right of the midline. A needle was inserted to a depth of 3 mm and withdrawn 0.4 mm to a depth of 2.6 mm. Two *μ*l of GL261-Luc2 cells (1–2 × 10^4^ cells/*μ*l) were infused over the course of 3 minutes. The burrhole was closed with bonewax and the incision was sutured. The mice were housed in groups of five in the animal care facility at St. Joseph’s Hospital and Medical Center in rooms with controlled temperature and humidity under a 12-hour light-dark cycle according to the guidelines outlined in the NIH Guide for Care and Use of Laboratory Animals^53^. Complete methods describing the GL261-Luc2 implantation into C57BL/6J mice have been previously published^16^.

### MR Imaging

Tumor bioluminescence and MR imaging were performed under isoflurane anesthesia on days 11, 15, 18, 22, and 25 after the initial intracranial injection. These imaging intervals were chosen to obtain frequent snapshots of the growing tumor in a manner that, in the experimentalists’ judgment, would not be detrimental to the health of the mice. For each imaging session, anesthesia was induced and maintained under isoflurane (1–2%) in oxygen. Respiration was continually monitored with a pillow sensor position under the abdomen (SA Instruments, Stony Brook, NY), and normal body temperature was maintained with a circulating warm water blanket (Thermo Scientific, Rockford, IL). The animals were stabilized with ear and tooth bars to minimize any motion and to ensure accurate image-to-anatomy registration. We present full details of the laboratory imaging here, although we have used only the T2-weighted images in this paper. Subsequent work will use this information to formulate more complex model frameworks.

Prior to MR imaging, bioluminescent tumor signal was measured. Animals received a subcutaneous injection of 150 *μ*g luciferin/kg body weight 15 min prior to *in vivo* imaging using an IVIS® Spectrum *in vivo* imaging system (Perkin Elmer, Waltham, MA). Images were acquired and analyzed using the system’s Living Image® 4.0 software.

MR images were acquired using a Bruker BioSpin 7T system in the following sequential order for each imaging session: high resolution T2-weighted (T2W), diffusion weighted (DW), T2W series, T1-weighted (T1W) series, dynamic contrast enhancement (DCE), and post contrast T1W. In the following discussion regarding imaging, we report the dimensions as axial × sagittal × coronal. High resolution T2W images (0.1 mm × 0.1 mm × 0.5 mm voxels) were acquired as a reference between time points. The DWI data series was acquired in 6 diffusion directions with a resolution of 0.2 mm × 0.2 mm × 1.0 mm. The T1W and T2W image series were acquired for T1 and T2 mapping, respectively. The resolution for these images was 0.2 mm × 0.2 mm × 0.5 mm. The DCE was continuously acquired for approximately 21 min, with the scientist needing to enter the MR room at 1.5 min into the acquisition to deliver a subcutaneous injection of a 1 ml bolus of contrast agent (i.e., Gd-DPTA (0.04 mol/ml)) through a preset catheter in the neck. The first 1.5 min of the DCE scan series was used as a pre-contrast baseline. The injections were completed approximately 2 min into the DCE acquisition. The resolution for these images was 0.2 mm × 0.2 mm × 0.5 mm. The full set of MRI parameters is provided in detail in Supplementary Data S1. Representative examples of the MR images are displayed in Fig. 1.

### Tissue Procurement and Histology

The mice were euthanized on day 26 using deep anesthesia with isoflurane followed by decapitation. The brains were carefully removed to minimize damage and were immediately placed in a 4% paraformaldehyde solution for 48 hours. The sample was then placed in a phosphate-buffered saline and submitted to St. Joseph’s Hospital and Medical Center’s histology lab for dehydration and paraffin embedding.

### Image Preprocessing

The brain, ventricle, and tumor spaces were segmented from the high resolution T2W images using the medical image processing software MIMICS® (Materials Inc, Leuven, Belgium). The segmented spaces were then exported as STL files, i.e., as surface meshes.

The brain surface meshes were used in two subsequent steps. First, the brain surface mesh from a given time point was imported back into MIMICS to identify and ultimately export point clouds of the intensity values included in all original images acquired during the same time point’s MR session. Second, brain meshes for a single animal at different time points were co-registered using the best-fit function in Geomagic® Studio (Geomagic Inc, NC). Specifically, the brain volumes were registered to the mid-time point, day 18. For each time point and for each mouse, Geomagic exported an affine matrix describing the necessary rotation, scaling, reflection, and shearing necessary to transform the surface of a given brain to the registered brain (which is the identity matrix for the third time point, day 18).

The exported affine matrices were then used to transform the corresponding resulting point clouds in MATLAB, so that the location of the brain for each mouse was constant. The transformed data points, containing intensity values, were then linearly interpolated to a uniform grid. The spacing of the grid is 0.1 mm × 0.1 mm × 0.5 mm to match the T2W image spacing, which allows for easy comparisons. This uniform grid is then used for the 3D finite difference and 2D level set methods described above.

### Availability of Data

The full datasets used in this article are available in the Open Science Framework Repository, https://osf.io/r78e4/.

### Availability of Computer Code

The 3D finite difference code used to generate the results for this article is available in the murine-GL261-tumors repository located on github, https://github.com/banderie/murine-GL261-tumors. The code is platform independent and written in MATLAB, which requires a license.

The 2D level set code used in this work may be made available by contacting Tracy L. Stepien. The code is platform independent and written in MATLAB, which requires a license.

## Electronic supplementary material

Dataset 1

Supplementary Figures

Supplementary Methods
