# Supplementary material for: Mathematical Analysis of Glioma Growth in a Murine Model
Source: Sci Rep. 2017 May 31;7:2508. doi: 10.1038/s41598-017-02462-0 (PMC5451439; doi:10.1038/s41598-017-02462-0)
Supplement: Supplementary file 2 — Supplementary Figures [file 41598_2017_2462_MOESM2_ESM.pdf]

Graphical Results for All 3D Simulations in Mathematical Analysis of Glioma Growth in a Murine Model

E. M. Rutter, T. L. Stepien, B. J. Anderies, J. D. Plasencia, E. C. Woolf, A. C. Scheck, G. H. Turner, Q. Liu, D. Frakes, V. Kodibagkar, Y. Kuang, M. C. Preul and E. J. Kostelich

The following contain full simulations results of the optimization for the 3D finite difference model under all hypotheses at all time points for each mouse. The simulation results of the optimization for the 3D finite difference model under Hypothesis 1 are shown in Figure 1 for Mouse 1, Figure 4 for Mouse 2 and Figure 7 for Mouse 3. Within each figure for Mouse 1 and Mouse 2, the subfigures a), b), c), and d) represent days 15, 18, 22, and 25 after injection. For Mouse 3, subfigures a) and b) represent days 22 and 25 after injection.

Figures 2, 5 and 8 are the results for Hypothesis 2 for Mouse 1, Mouse 2 and 3, respectively, and Figures 3 6 and 9 are the results for Hypothesis 3 for Mouse 1,Mouse 2 and 3, respectively.

The results are similar to the Mouse 1 results in the main paper: Hypothesis 1 generates the worst fit, while Hypothesis 3 provides the best fit.

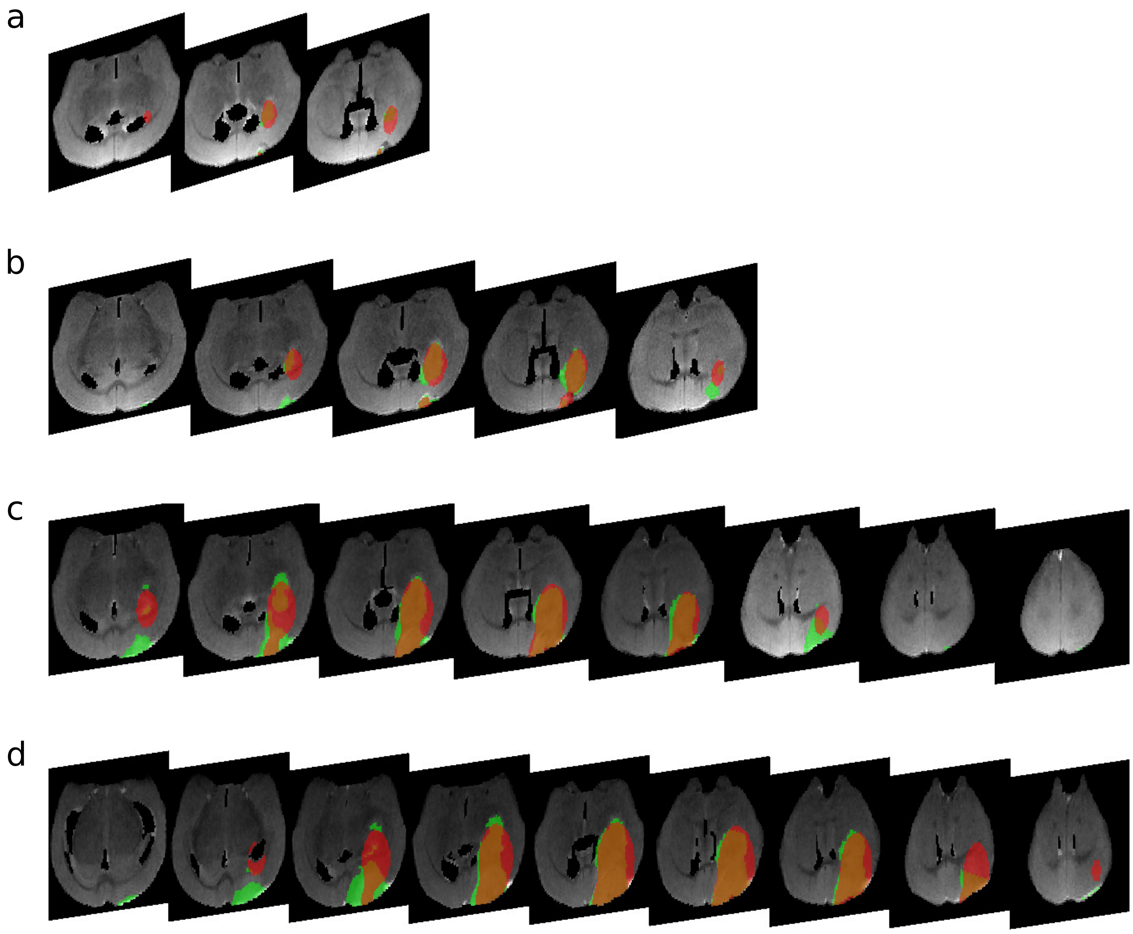

Supplementary Fig. 1: Representative slices for Mouse 1 under Hypothesis 1. The subfigures show individual time fits: (a) Time point 2 (day 14); (b) time point 3 (day 22); (c) time point 4 (day 22); (d) time point 5 (day 25). Simulated tumor is highlighted in red and actual tumor is green.

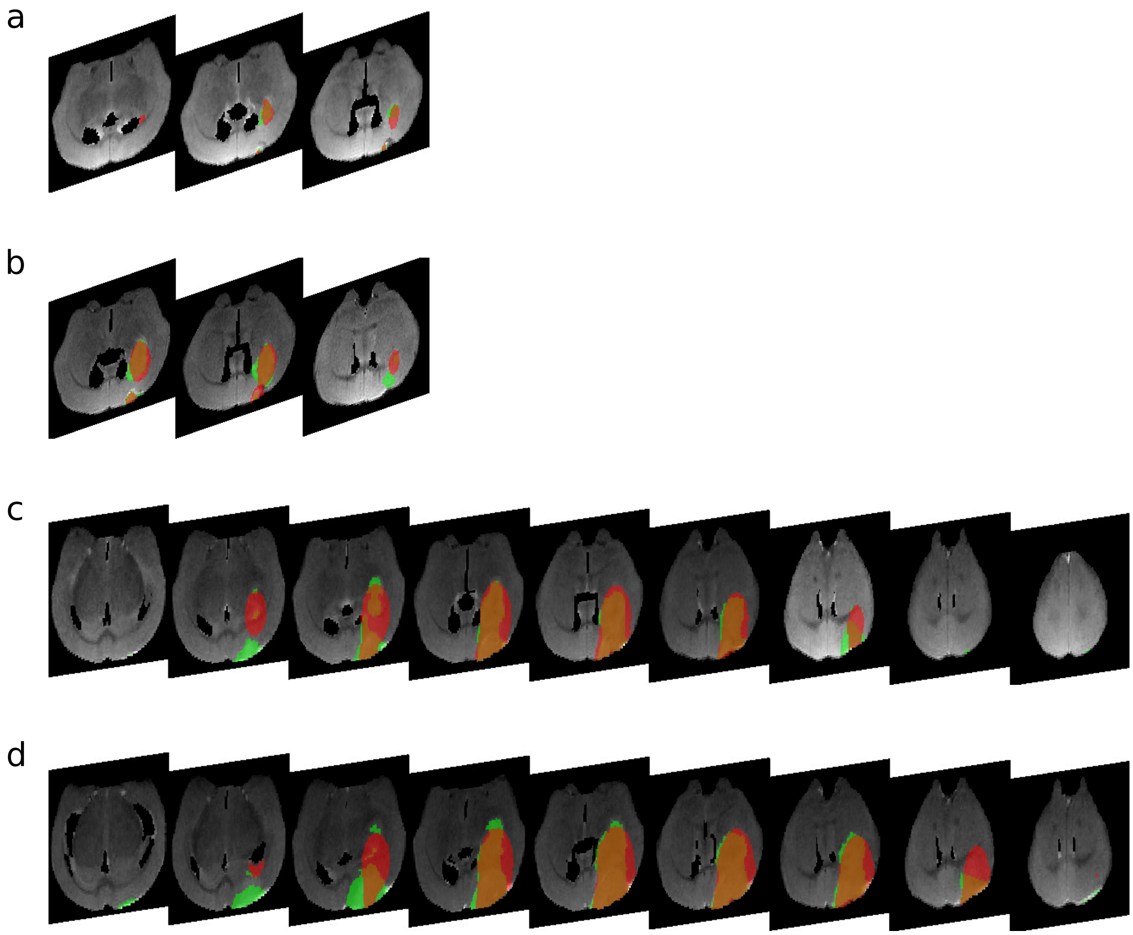

Supplementary Fig. 2: Representative slices for Mouse 1 under Hypothesis 2. The simulations are otherwise the same as in Supplementary Fig. 1.

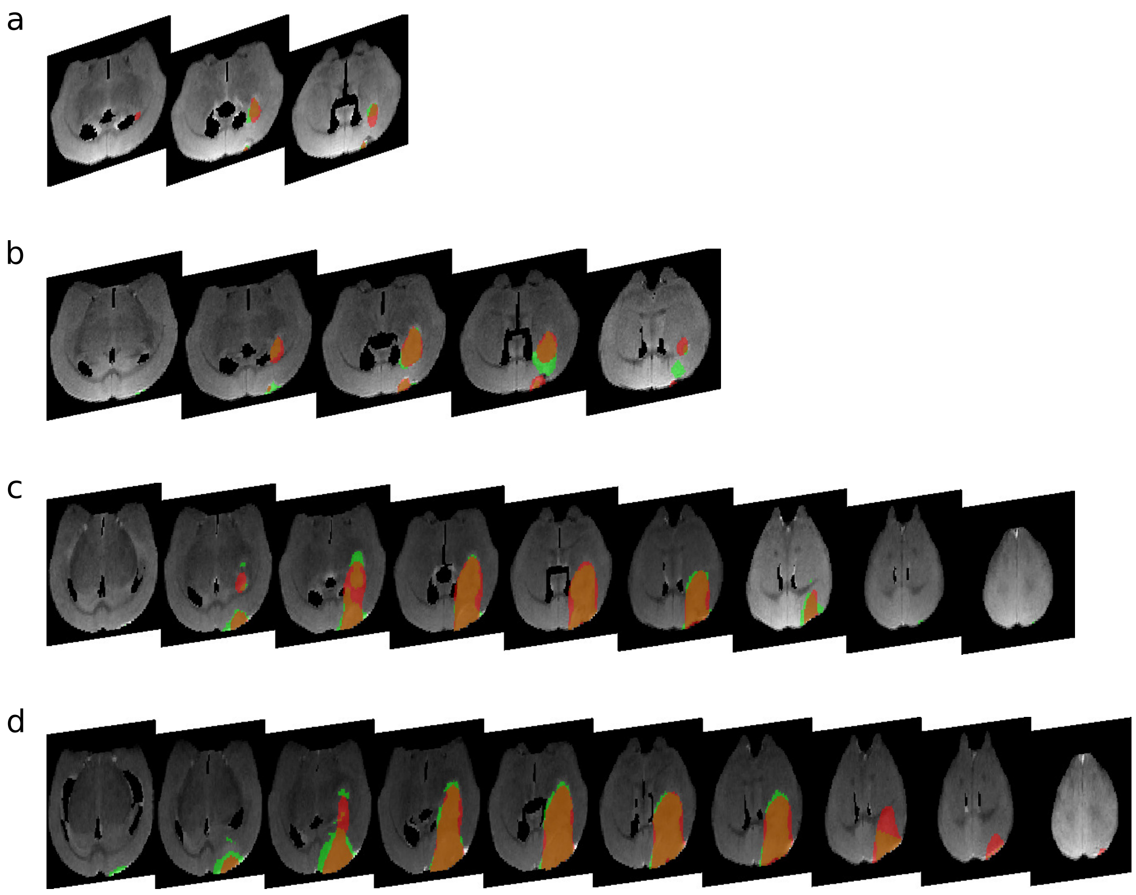

Supplementary Fig. 3: Representative slices for Mouse 1 under Hypothesis 3. The simulations are otherwise the same as in Fig. 1.

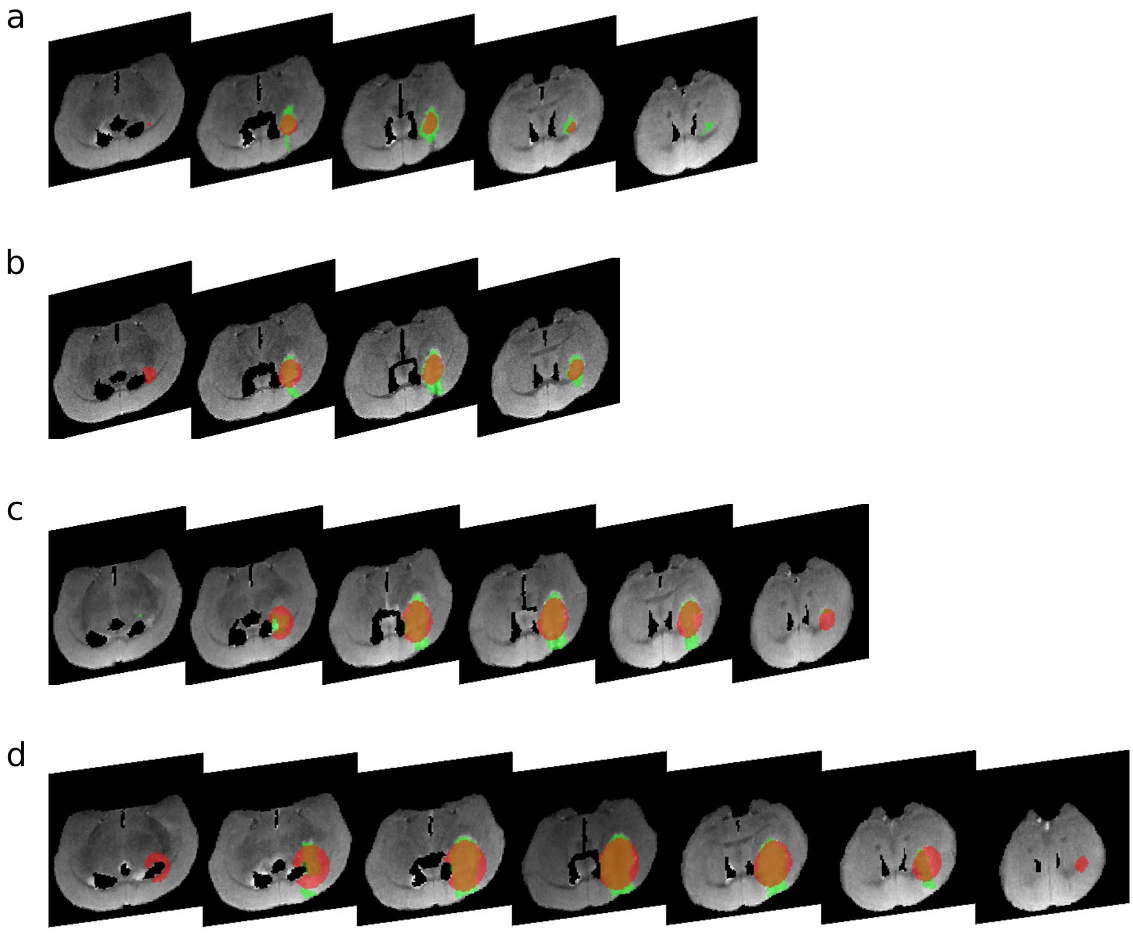

Supplementary Fig. 4: Representative slices for Mouse 2 under Hypothesis 1. The simulations are otherwise the same as in Fig. 1.

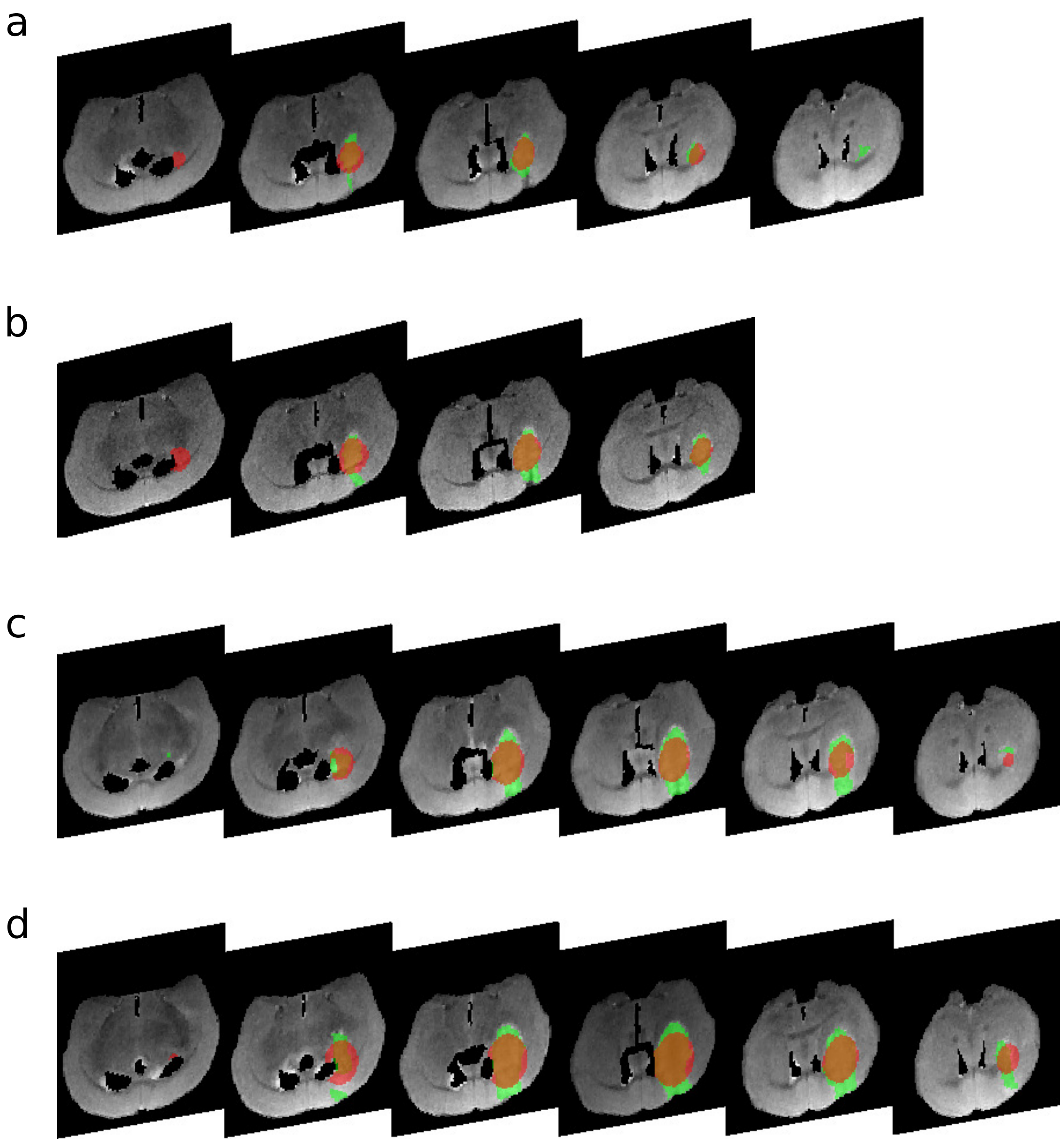

Supplementary Fig. 5: Representative slices for Mouse 2 under Hypothesis 2. The simulations are otherwise the same as in Fig. 1.

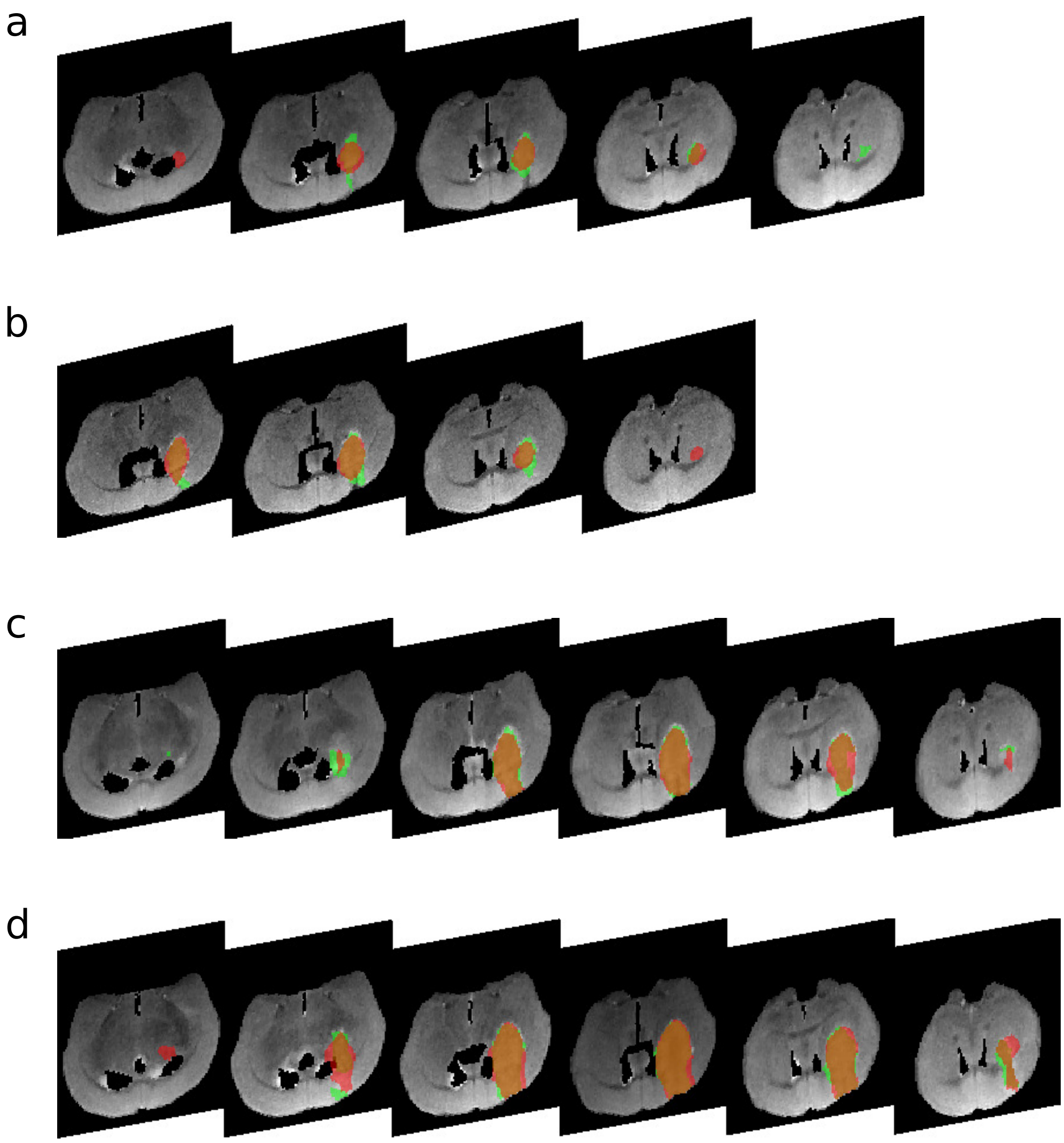

Supplementary Fig. 6: Representative slices for Mouse 2 under Hypothesis 3. The simulations are otherwise the same as in Fig. 1.

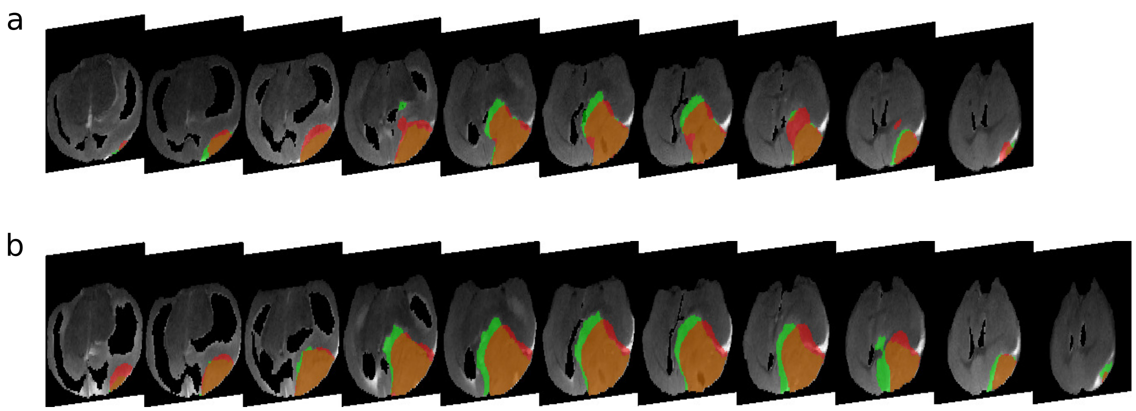

Supplementary Fig. 7: Representative slices for Mouse 3 under Hypothesis 1. We use time point 3 (day 18) as the initial condition, so the subfigures are now (a) time point 4 (day 22) and (b) time point 5 (day 25). The simulations are otherwise the same as in Fig. 1.

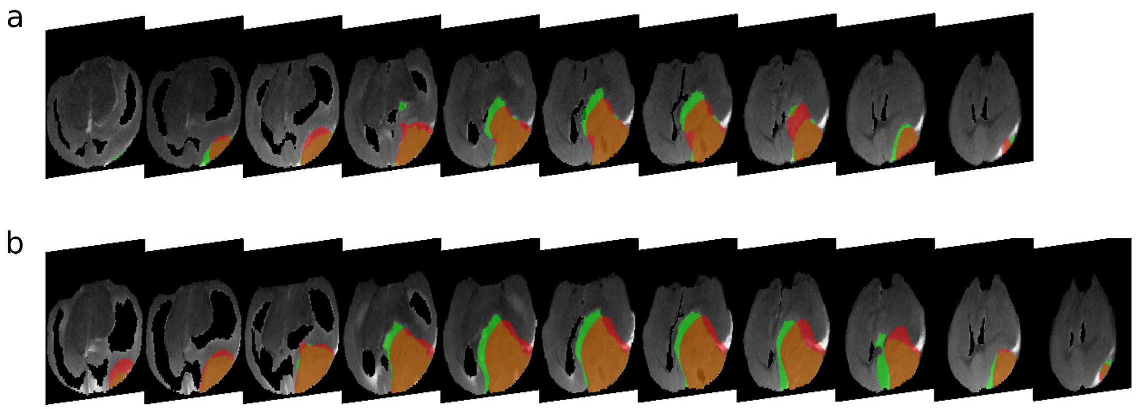

Supplementary Fig. 8: Representative slices for Mouse 3 under Hypothesis 2. We use time point 3 (day 18) as the initial condition, so the subfigures are now (a) time point 4 (day 22) and (b) time point 5 (day 25).The simulations are otherwise the same as in Fig. 1.

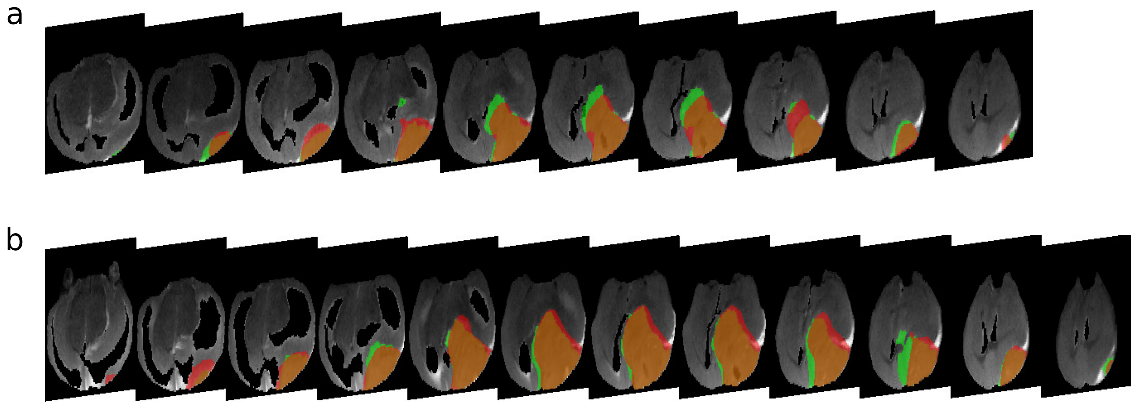

Supplementary Fig. 9: Representative slices for Mouse 3 under Hypothesis 3. We use time point 3 (day 18) as the initial condition, so the subfigures are now (a) time point 4 (day 22) and (b) time point 5 (day 25).The simulations are otherwise the same as in Fig. 1.
