# Supplementary material for: Mathematical Analysis of Glioma Growth in a Murine Model
Source: Sci Rep. 2017 May 31;7:2508. doi: 10.1038/s41598-017-02462-0 (PMC5451439; doi:10.1038/s41598-017-02462-0)
Supplement: Supplementary file 3 — Supplementary Methods [file 41598_2017_2462_MOESM3_ESM.pdf]

2D Level Set Methodology in Mathematical Analysis of Glioma Growth in a Murine Model

E. M. Rutter, T. L. Stepien, B. J. Anderies, J. D. Plasencia, E. C. Woolf, A. C. Scheck, G. H. Turner, Q. Liu, D. Frakes, V. Kodibagkar, Y. Kuang, M. C. Preul, and E. J. Kostelich

For the study of glioblastoma tumor growth in two-dimensional slices of brain sections, the Stefan problem is solved numerically using a level set method. The level set method was introduced by Osher and Sethian [1] and applied to Stefan problems by Chen et al. [2], Javierre et al. [3], Arciero et al. [4], among others.

The level set method tracks a moving boundary (defined as the boundary of the tumor cells in this context), which is represented as the zero level set of a smooth function Φ on a fixed grid. In the schematic diagram in Figure 5 in the main text, the moving boundary of the tumor cells is $\partial\Omega_1^t$. Initially, this level set function describes the signed distance d from each grid point to the boundary, where Φ is positive if there are cells in that location, zero on the boundary, or negative otherwise. In other words,

$$\Phi(\mathbf{x},0) = \begin{cases} d(\mathbf{x},\partial\Omega_1^0), & \mathbf{x}\in\Omega_1^0 \\ 0, & \mathbf{x}\in\partial\Omega_1^0 \\ -d(\mathbf{x},\partial\Omega_1^0), & \mathbf{x}\notin\Omega_1^0. \end{cases} \tag{1}$$

In our implementation, the distance from any grid node to a boundary is calculated as the minimum Euclidean distance from the grid node position to all the boundary position coordinates.

The first step of the algorithm involves moving the level set function Φ with velocity determined by the Stefan condition, equation 7 in the main text. The velocity $\mathbf{v} = (\upsilon^x, \upsilon^y, \upsilon^\eta, \upsilon^\zeta)$ for the boundary is computed, respectively, in the standard Cartesian coordinates x, y and the 45°-rotated coordinates η and ζ, since the four coordinate directions reduce grid orientation effects [2].

The velocity components are then continuously extended off the boundary to the entire domain for each layer via the advection equations

$$\frac{\partial \upsilon^x}{\partial \tau} + \mathrm{sign}(\Phi\Phi_x)\frac{\partial \upsilon^x}{\partial x} = 0, \tag{2a}$$

$$\frac{\partial \upsilon^y}{\partial \tau} + \mathrm{sign}(\Phi\Phi_y)\frac{\partial \upsilon^y}{\partial y} = 0, \tag{2b}$$

$$\frac{\partial \upsilon^\eta}{\partial \tau} + \mathrm{sign}(\Phi\Phi_\eta)\frac{\partial \upsilon^\eta}{\partial \eta} = 0, \tag{2c}$$

$$\frac{\partial \upsilon^{\zeta}}{\partial \tau} + \text{sign}(\Phi \Phi_{\zeta}) \frac{\partial \upsilon^{\zeta}}{\partial \zeta} = 0, \tag{2d}$$

where $\upsilon^*(\mathbf{x}, 0) = -\frac{D}{\bar{\rho}} \frac{\partial \rho}{\partial *}$ for $* = x, y, \eta, \zeta$, and τ is a pseudo-time. These equations are discretized with a first-order upwind scheme, and if a grid point is too close to the boundary, an extended neighborhood near that point is used to determine the velocity [2].

Once the components of the (now continuously extended) velocity $\mathbf{v}$ have been obtained, we propagate the level set function via

$$\frac{\partial \Phi}{\partial t} + \mathbf{v} \|\nabla \Phi\| = 0, \tag{3}$$

which is solved using a forward Euler discretization in time and weighted essentially non-oscillatory (WENO) approximations to the spatial derivatives.

The level set function in general ceases to be an exact distance function, i.e., $\|\nabla \Phi\| \neq 1$, even after one time step [5]. To avoid steep or flat gradients in Φ near the moving boundaries, the level set functions are reinitialized to be exact distance functions from the moving boundary at every time step. Given a function Φ_0 that is not an exact distance function, we can evolve it into an exact distance function by iterating the following to steady state

$$\frac{\partial \Phi}{\partial \tau} = \overline{\text{sign}}(\Phi_0)(1 - \|\nabla \Phi\|), \tag{4a}$$

$$\Phi(\mathbf{x}, 0) = \Phi_0(\mathbf{x}). \tag{4b}$$

Here τ is a pseudo-time and $\overline{\text{sign}}$ is the smooth sign function $\overline{\text{sign}}(x) = x/\sqrt{x^2 + \epsilon^2}$. The equation is discretized using Godunov's method in pseudo-time, a third-order Runge-Kutta scheme in real time, and fifth-order WENO approximations for the spatial gradient. Only 3–10 iterations are needed for sufficient accuracy to evolve Φ to an exact distance function [3].

After the level set function Φ has moved the correct velocity at the moving boundary and been reinitialized as an exact distance function, we solve equation 1 from the main text for the density of the tumor cells over the entire domain using a finite difference scheme with adjustments for grid nodes near the boundary and the backward Euler method for time integration.

Away from the moving boundary, the standard 5-point stencil scheme is used for the Laplacian. For grid nodes that border the moving boundaries, the scheme is adjusted using interpolating polynomials and one-sided differencing with values of Φ near the interface and the boundary conditions, Equations 6–7 of the main text [2]. For grid nodes that are outside of the domain Ω_2^t, a cut-cell method is used to adapt the grid by "cutting" grid nodes that are not located within the domain [3].

A numerical implementation of this level set method was developed by Javierre et al. [3], adapted by Arciero et al. [4] for wound healing and cell colony growth, and then adapted here to produce the numerical solutions in this study.

References

[1] Osher S, Sethian JA. Fronts propagating with curvature-dependent speed: algorithms based on Hamilton-Jacobi formulations. J Comput Phys. 1988;79:12–49.

[2] Chen S, Merriman B, Osher S, Smereka P. A simple level set method for solving Stefan problems. J Comput Phys. 1997;135:8–29.

[3] Javierre E, Vuik C, Vermolen FJ, Segal A. A level set method for three dimensional vector Stefan problems: Dissolution of stoichiometric particles in multi-component alloys. J Comput Phys. 2007;224:222–240.

[4] Arciero JC, Mi Q, Branca MF, Hackam DJ, Swigon D. Continuum model of collective cell migration in wound healing and colony expansion. Biophys J. 2011;100:535–543.

[5] Sussman M, Smereka P, Osher S. A level set approach for computing solutions to incompressible two-phase flow. J Comput Phys. 1994;114:146–159.
